# *Acinetobacter lactucae* Strain QL-1, a Novel Quorum Quenching Candidate Against Bacterial Pathogen *Xanthomonas campestris* pv. *campestris*

**DOI:** 10.3389/fmicb.2019.02867

**Published:** 2019-12-17

**Authors:** Tian Ye, Tian Zhou, Xinghui Fan, Pankaj Bhatt, Lianhui Zhang, Shaohua Chen

**Affiliations:** ^1^State Key Laboratory for Conservation and Utilization of Subtropical Agro-Bioresources, Integrative Microbiology Research Centre, Guangdong Province Key Laboratory of Microbial Signals and Disease Control, South China Agricultural University, Guangzhou, China; ^2^Guangdong Provincial Laboratory of Lingnan Modern Agricultural Science and Technology, South China Agricultural University, Guangzhou, China

**Keywords:** quorum sensing, diffusible signal factor, *Acinetobacter lactucae*, biocontrol, *Xanthomonas campestris* pv. *campestris*

## Abstract

Quorum sensing (QS) is a cell–cell communication mechanism among bacterial populations that is regulated through gene expression in response to cell density. The pathogenicity of *Xanthomonas campestris* pv. *campestris* (*Xcc*) is modulated by the diffusible signal factor (DSF)-mediated QS system. DSF is widely conserved in a variety of gram-negative bacterial pathogens. In this study, DSF-degrading bacteria and their enzymes were thoroughly explored as a biocontrol agent against *Xcc*. The results indicated that a novel DSF-degrading bacterium, *Acinetobacter lactucae* QL-1, effectively attenuated *Xcc* virulence through quorum quenching. Lab-based experiments indicated that plants inoculated with QL-1 and *Xcc* had less tissue decay than those inoculated with *Xcc* alone. Co-inoculation of strains *Xcc* and QL-1 significantly reduced the incidence and severity of disease in plants. Similarly, the application of crude enzymes of strain QL-1 substantially reduced the disease severity caused by *Xcc*. The results showed that strain QL-1 and its enzymes possess promising potential, which could be further investigated to better protect plants from DSF-dependent pathogens.

## Introduction

Cruciferous vegetables are of global economic importance and include a wide range of unique members such as Bok Choy, cabbage, and broccoli (Rakow, 2004; Somec and Sondi, 2019). Black rot caused by *Xanthomonas campestris* pv. *campestris* (*Xcc*) becomes severely destructive under warm and humid conditions (Gupta et al., 2013). This leads to serious economic losses by reducing the shelf life and market value of infected crop plants (Massomo, 2002; Massomo et al., 2004). To overcome the effects of black rot, chemical control (Mew and Natural, 1993; Mishra and Arora, 2012; Vicente and Holub, 2013), cultivation of certified disease-free transplants and seeds, cultivation of resistant cultivars, hot water treatment (Nega et al., 2003), and drip irrigation are generally recommended (Luiz et al., 2016). Over the past few decades, pesticides have been the main preventative applied against black rot. Pesticide overuse results in increased production cost, pesticide-resistant strains, human health issues, and environmental concerns (Sexton et al., 2007). Therefore, biological control by quorum quenching (QQ) has been focused on in recent years as a promising alternative strategy against plant disease (Bzdrenga et al., 2017). QQ disrupts quorum sensing (QS) either by the degradation of QS signals or interference with signal generation or perception (He and Zhang, 2008).

Quorum sensing is a common density-dependent mode of communication between bacteria. The expression of QS-dependent genes is regulated by synthesizing and secreting signal molecules and auto-inducers (AIs) (Miller and Bassler, 2000). Signal molecules accumulate in the extracellular environment and are recognized by bacteria to detect their own population density. When the population density reaches a certain threshold, the signal molecule binds to the corresponding receptor protein and activates the expression of downstream-related genes (Fuqua and Winans, 1994). Many gram-negative bacteria have QS systems to synchronize their behavior and monitor population density-dependent growth (Nealson and Hastings, 1979). QS-regulated pathways play a central part in biofilm formation and the secretion of virulence factors for pathogenicity, toxicity, surface-attachment, and antimicrobial agent resistance (Kaye and Pogue, 2015).

Quorum quenching is a reverse technique that targets QS to affect the growth of pathogenic bacteria. QQ using enzymes and QS-inhibitors can be conducted without affecting bacterial growth. Effective reduction of bacterial infections without inducing resistance makes QQ an attractive strategy to control plant disease (Kalia, 2015). QQ enzymes are especially promising because of their extracellular AI degradation and because they can be applied in large quantities. QQ enzymes can be applied in various sectors including human health, aquaculture, agriculture, water supply and drainage, and biofouling (Bzdrenga et al., 2017). Currently, the most commonly discovered enzymes are *N*-acyl homoserine lactone (AHL)-degrading enzymes, including AHL-lactonase, AHL-acylase, and oxidoreductases (Dong and Zhang, 2005; Waters and Bassler, 2005; Krysciak et al., 2011). *Bacillus* sp. has been reported to naturally produce lactonases or acylases to degrade AHL signals (Dong et al., 2000; Zhang et al., 2002; Carlier et al., 2003). Degradation of AHLs by *Bacillus thuringiensis* lactonase (AiiA) reduced *Pectobacterium carotovorum* pathogenicity on potato slices (Dong et al., 2004). Quenching signaling molecules to disrupt the QS regulatory function of bacteria is a feasible strategy for controlling signaling-mediated diseases.

The quorum-sensing signal of *Xcc* is known as DSF and has been characterized as an α and β unsaturated fatty acid, *cis*-11-methyl-2-dodecenoic acid (Supplementary Figure S1; Wang et al., 2004; He and Zhang, 2008). DSF represents a family of widely conserved QS signals involved in the regulation of virulence factor production in a variety of gram-negative bacteria (Zhou et al., 2017). DSF plays a key role in regulating virulence, motility, antibiotic resistance, biofilm formation, and interspecies and inter-kingdom communications (Boon et al., 2008; Deng et al., 2011).

In this study, a highly efficient DSF-degrading *Acinetobacter lactucae* strain QL-1 was identified. The DSF-degrading ability, degradation products, and degradation mechanism of QL-1 were studied. Furthermore, the properties of QL-1 and its crude enzymes were investigated to develop pre-infection preventive measures against DSF-dependent bacterial pathogens. This preliminary study of QL-1 as a biological control agent provides new insight for signaling-mediated plant disease control.

## Materials and Methods

### Chemicals and Plants

Diffusible signal factor (≥99%) was purchased from Shanghai UDChem Technology Co., Ltd. The pure active ingredient of pesticide, streptomycin (STR) (98%), was used as a positive control at 3.3 g mL^–1^. Radish (*Raphanus sativus*) was purchased from a local market (Guangzhou, China), and healthy plants were selected for the experiments.

### Strains and Culture Conditions

*Xanthomonas campestris* pv. *campestris* XC1 and *Escherichia coli* DH5α were provided by the Integrative Microbiology Research Centre, South China Agricultural University, China. *Xcc* strains were maintained in LB [composition (g L^–1^): yeast extract 5.0, tryptone 10.0, NaCl 10.0] with rifampicin (RIF) (30 μg mL^–1^) at 30°C, whereas *E. coli* strains were grown in LB at 37°C.

### Isolation and Screening of Bacteria

Soil samples were collected from Guangzhou, China, for the isolation of bacteria. Approximately 300 g of soil was collected from the upper layer (10–20 cm), and samples were stored at 4°C after collection. To isolate bacteria, 5 g of soil samples were added to minimal salt medium (MSM) [composition (g L^–1^): [NH_4_]_2_SO_4_ 2.0, Na_2_HPO_4_⋅12H_2_O 1.5, KH_2_PO_4_ 1.5, MgSO_4_⋅7H_2_O 0.2, CaCl_2_⋅2H_2_O 0.01, FeSO_4_⋅7H_2_O 0.001, pH 7.2] supplemented with 50 μmol L^–1^ DSF and cultivated for 7 days at 30°C and 200 rpm. After 7 days, the suspension was transferred to fresh MSM containing DSF (100 μmol L^–1^) at 10% inoculum and cultivated under the same conditions for 7 days. This procedure was repeated until the DSF concentration increased to 400 μmol L^–1^. The final suspension was serially diluted (10^–1^–10^–8^) and spread on LB agar plates. After incubation at 30°C for 24–72 h, colonies with different characteristics were selected and transferred to fresh medium (Yang et al., 2018). The procedure was repeated until a pure culture was obtained.

To screen, bacterial isolates were grown in MSM containing 1 mmol L^–1^ DSF as the sole source of carbon for 24 h at 30°C and 200 rpm. After 24 h, cultures were centrifuged, and the remaining DSF was extracted from the supernatant. Initially, culture supernatant was extracted three times with an equal volume of ethyl acetate (Deng et al., 2010). Then, the organic phase was evaporated in a rotary evaporator until dried and was then finally dissolved in 2 mL methanol. The remaining amount of DSF was determined by high-performance liquid chromatography (HPLC).

### Screening of Isolates for Better DSF-Degradation Activity

The morphology of the selected isolates was studied by inoculating them on LB medium for 24 h at 30°C. To conduct phylogenetic analysis, the 16S rRNA gene was amplified with forward primer 27F (5′-AGAGTTTGATCCTGGCTCA-3′) and reverse primer 1492R (5′-GGTTACCTTGTTACGACTT-3′) (Tang et al., 2013). The PCR conditions were as follows: 5 min of denaturation at 94°C, followed by 33 cycles at 94°C for 30 s, 55°C for 30 s, and a final extension at 72°C for 60 s. The purified PCR product was sequenced by Shanghai Invitrogen Technology Co. Ltd., China. Similarity analysis of 16S rDNA was conducted with the Basic Local Alignment Search Tool (BLAST) of the National Center for Biotechnology Information (NCBI) database. Closely similar sequences were used for the identification and phylogenetic analysis of the isolated strain. The Guangdong Detection Center of Microbiology was commissioned to perform biological tests.

### Inhibition Test

Interactions between strain QL-1 and pathogen XC1 were studied on LB plates according to the method of Garge and Nerurkar (2017). The purpose of this was to study virulence attenuation of *Xcc* through DSF degradation and not antagonistic activity. Strain QL-1 and pathogen XC1 were inoculated into 5 mL LB medium and incubated overnight at 30°C and 200 rpm. Concentrations of strain QL-1 and the pathogen XC1 were determined by ultraviolet spectrophotometer at 600 nm (OD_600_). Then, 5 mL of overnight XC1 suspension (OD_600_ = 1) was added into agar containing LB medium (at about 50°C) and thoroughly mixed. The mixture was immediately poured into plates (about 20 mL/plate). A sterilized cork borer was used to punch holes in the plates, and 10 μL of overnight QL-1 suspension (OD_600_ = 1) was injected into the holes. The inhibition zone was observed after 24 h of incubation (Garge and Nerurkar, 2017). Each treatment was carried out with three replicates, and the entire experiment was conducted five times.

### DSF Degradation Test

To determine the relationship between the DSF degradation rate and the growth of strain QL-1, 2 mmol L^–1^ DSF was added to isolate suspensions, and the amount of DSF remaining after different intervals was determined using HPLC. Initially, QL-1 was inoculated into 5 ml LB medium and incubated overnight at 30°C and 200 rpm. Overnight cultures and 2 mmol L^–1^ DSF were added to MSM and incubated for 24 h at 30°C and 200 rpm, whereas MSM containing 2 mmol L^–1^ DSF served as control. Subsequently, the remaining DSF was extracted as described above and quantified. The growth of QL-1 at different intervals was measured using an ultraviolet spectrophotometer.

### Identification of Degradation Products by GC–MS

To identify DSF and its degradation products, QL-1 was grown in MSM containing 5 mmol L^–1^ DSF. Non-inoculated samples containing the same amount of DSF served as controls. Samples of different treatments were collected and extracted at regular intervals, as described above. Samples were analyzed by Agilent Mass-Hunter Workstation software and a gas chromatography–mass spectrometry (GC–MS) (Model 7890B/5977B, Agilent Technologies, United States). Data acquisition was carried out in scan mode. The GC inlet was at 250°C and was operated in split mode at a 10:1 ratio. All analyses were performed with an Agilent HP-5MS Ultra Inert column (30 m × 0.25 μm × 0.25 μm). During irradiation, the GC oven was kept at 60°C to prevent column damage. Temperatures were maintained as follows: 60°C isothermal for 5 min; 10°C/min up to 300°C, and then isothermal for 29 min. The transfer-line was kept at 280°C, and mass spectra were recorded in the *m*/*z* range 40–430 (Chen et al., 2015). To identify the degradation products, mass spectrometry analyses were compared with the authentic standard compounds of the National Institute of Standards and Technology (NIST, United States) library database.

### Preparation of Crude Enzymes

A loopful of QL-1 culture was inoculated into 10 mL LB medium and incubated overnight at 30°C and 200 rpm. Cells were harvested by centrifugation (10,000 rpm for 10 min at 4°C), and the supernatant was used to detect extracellular enzymes. Cells were washed thrice with phosphate buffer solution (PBS) (pH 7.4) and re-suspended in PBS (Hsiao et al., 2011). Cells were disrupted by sonication at 4°C and 20 amplitude, and 30 strokes were conducted over an interval of 5 s. The homogenate was centrifuged under cold conditions at 10,000 rpm for 10 min, and clear supernatant was collected for use as a source of intracellular enzymes.

### Effect of the Isolate Against *Xcc*

Experiments were carried out in an artificial climate box to evaluate the suppressive and preventive effect of the isolate against *Xcc* by following the modified method of Ryan et al. (2007). Healthy radish plants were thoroughly washed with tap water and sterilized. To sterilize the surface, plants were sequentially immersed in 44% sodium hypochlorite and 70% ethanol for 60 s. Finally, plants were rinsed with sterile distilled water (D/W), and this cycle was repeated three times. Sterilized plants were cut into slices (4–5 mm thick). The experimental design included seven treatments as follows: (1) untreated plant slices, serving as control; (2) plant slices treated with QL-1 (1 × 10^9^ CFU mL^–1^); (3) plant slices treated with XC1 (4 × 10^8^ CFU mL^–1^); (4) plant slices treated with QL-1 (1 × 10^9^) and XC1 (4 × 10^8^ CFU mL^–1^); (5) plant slices treated with *E. coli* DH5α (8 × 10^8^ CFU mL^–1^) and XC1 (4 × 10^8^ CFU mL^–1^); (6) plant slices treated with agricultural STR and XC1 (4 × 10^8^ CFU mL^–1^); and (7) inoculation of QL-1 (1 × 10^9^ CFU mL^–1^) on radish slices followed by pathogen XC1 (4 × 10^8^ CFU mL^–1^) after 6 h. All treatments consisted of three replicates, and the experiments were repeated five times. Disease severity was evaluated according to the macerated area and macerated tissue weight. The diameter of the macerated region (square millimeters) was measured and scooped out to measure the weight. The percentage of maceration was calculated by comparing with the pre-inoculation tissue weight.

### Effect of QL-1 Crude Enzymes Against *Xcc*

The crude enzyme of strain QL-1 was applied against *Xcc*. The experimental design included four treatments: (1) untreated plant slices, serving as control; (2) plant slices treated with XC1 at 4 × 10^8^ CFU mL^–1^; (3) plant slices treated with 1 mL extracellular enzymes of QL-1 and XC1 at 4 × 10^8^ CFU mL^–1^; and (4) plant slices treated with 1 mL intracellular enzymes of strain QL-1 and XC1 at 4 × 10^8^ CFU mL^–1^. All treatments consisted of three replicates, and experiments were repeated five times. Disease severity was evaluated as described above.

### Antimicrobial Susceptibility Test

The antimicrobial susceptibility of QL-1 was also tested. QL-1 was inoculated into 5 ml LB medium and incubated overnight at 30°C and 200 rpm. Overnight cultures were added to LB medium supplemented with different concentrations of various antibiotics and incubated for 24 h at 30°C and 200 rpm. Antibiotics included chloramphenicol (CM, 30 mg mL^–1^), ampicillin (AMP, 50 mg mL^–1^), RIF (25 mg mL^–1^), STR (50 mg mL^–1^), carbenicillin (CARB, 50 mg mL^–1^), kanamycin (KAN, 50 mg mL^–1^), gentamicin (GEN, 50 mg mL^–1^), and tetracycline (TET, 5 mg mL^–1^). Ten concentrations (5, 10, 20, 50, 150, 200, 250, 300, 350, and 400 g mL^–1^) of each antibiotic were used in this experiment. The growth of QL-1 in the different antibiotic concentrations was detected by ultraviolet spectrophotometer at 600 nm. All treatments were carried out in triplicate and were repeated three times.

### Statistical Analysis

The experimental data were analyzed by one-way analysis of variance (ANOVA), and means were compared by Bonferroni’s multiple comparison test in Graphpad Prism (Version 6). Experiments were arranged according to a completely randomized design, and *P*-values < 0.05 were considered statistically significant.

## Results

### Selection of Strain QL-1 as Rapidly Degrading DSF

In the present study, a simple and efficient method was developed to effectively screen DSF-degrading microorganisms. Bacterial strains from various soil samples were enriched in MSM containing 50 μmol L^–1^ of DSF as the sole carbon source. According to the results of soil enrichment culture, several morphologically different bacterial strains producing clear zones on MSM plates supplemented with DSF were isolated from various soils. Strains were screened on the basis of their DSF degradation potential. Strain QL-1 rapidly degraded DSF and was selected for further studies.

### Morphological Characterization and Identification of Strain QL-1

**FIGURE 1:**
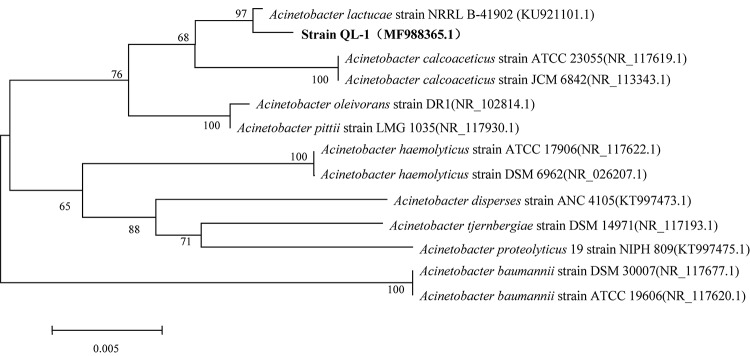
Phylogenetic tree using 16S rRNA sequences of *Acinetobacter lactucae* QL-1 and related strains. Numbers in parentheses represent sequence accession numbers in GenBank. Numbers at the nodes indicate bootstrap values from neighborhood-joining analysis of 1000 re-sampled data sets. Bar represents sequence divergence.

QL-1 utilized D-glucose, D-fucose, L-arginine, L-alanine, and L-lactic acid but was unable to utilize D-lactose, sucrose, glycerin, or D-mannitol (Supplementary Table S1). When grown on blood plates, strain colonies appeared milky white, small, round, convex, and smooth with a translucent surface and clear edges (Supplementary Figure S2). The morphology of QL-1 was observed to be that of gram-stain-negative rods that formed cocci of approximately 0.7–1.5 × 0.8–1.0 μm in the late stationary phase (Supplementary Figure S3). Cells paired to form chains of different lengths. The cellular and colony morphology of the strain was similar to that of *A. lactucae* strain NRRL B-41902 (Rooney et al., 2016). NCBI BLAST analysis of the 16S rRNA sequence revealed a high similarity (≥98%) of strain QL-1 with different *A. lactucae* strains. The phylogenetic tree of the 16S rRNA sequence indicated that strain QL-1 was in the *Acinetobacter* group (Figure 1). The bacterium was further confirmed (99%) as *A. lactucae* by way of Biolog Identification Systems software. Based on morphological, biochemical, and molecular characterization and Biolog testing, strain QL-1 was identified as *A. lactucae* (accession number MF988365.1).

### QL-1 Showed No Antagonistic Activity Against *Xcc*

In this study, the results of inhibition testing confirmed that no inhibition zone occurred when QL-1 and *Xcc* grew together, which means that QL-1 showed no antagonism against *Xcc* (Supplementary Figure S4). The study also indicated that virulence attenuation of *Xcc* by QL-1 was through DSF degradation and not through antagonistic activity. Therefore, this strain was selected for further studies.

### DSF Degradation Kinetics

**FIGURE 2:**
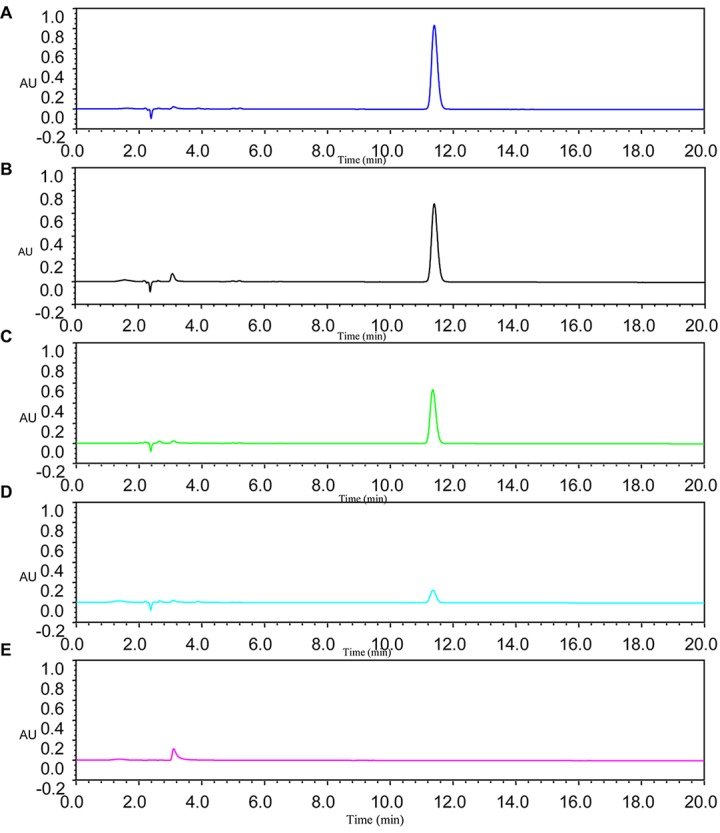
Remaining amount of DSF at different time intervals was determined by HPLC. Panel **A** was the MSM with DSF alone as control; Panels **B–E** represent DSF degradation by strain QL-1 at 6, 9, 12, and 15 h, respectively.

**FIGURE 3:**
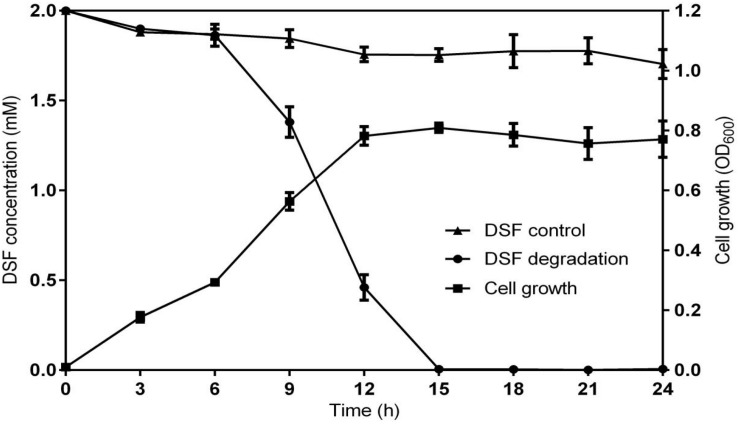
Relationship between DSF degradation and growth of QL-1. Values represent the mean of three repeats. Each experiment was conducted with three replicates. Bars indicate standard deviation of the mean. Symbols: ▲, DSF control; ∙, DSF degradation by strain QL-1; and ■, cell growth.

Diffusible signal factor degradation was as 7.3, 34.1, 78.5, and 100% after 6, 9, 12, and 15 h, respectively. The remaining amount of DSF at different time intervals is shown in Figure 2. During the fastest growing period (0–15 h), the OD_600_ reached 0.820, and the DSF concentration decreased from 2.0 to 0 mmol L^–1^. DSF was completely degraded within 15 h, and DSF degradation was positively correlated with cell growth (Figure 3). Degradation in the control was significantly lower and reached only 10% after 15 h due to biotic factors.

### Identification of Degradation Products by GC–MS

**FIGURE 4:**
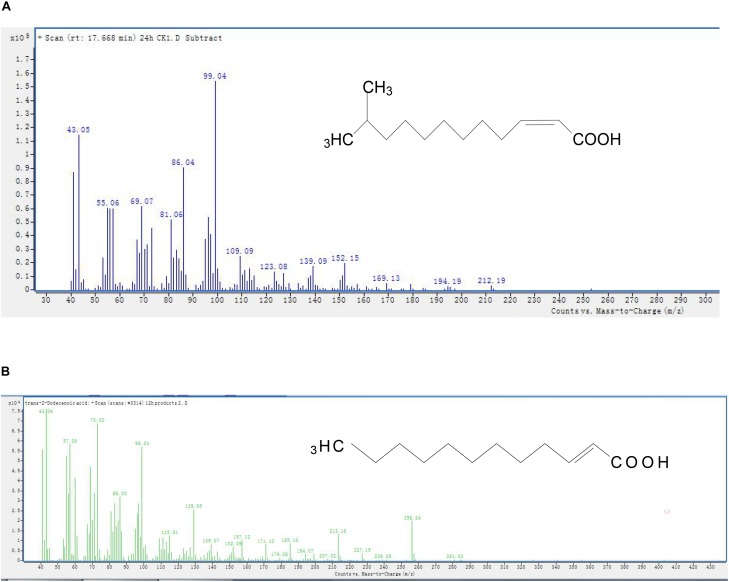
Mass spectra and proposed structures of detected degradation products identified with GC. Peaks 1 and 2 in Supplementary Figure S5: **(A)** DSF; **(B)** *trans*-2-dodecenoic acid.

The GC peaks of degradation products are shown in Supplementary Figure S5. After background correction, the mass spectra of each chromatographic peak were compared with the control and installed libraries of mass spectra and were separately recounted. In comparison to the control, unmarked peaks were identified as impurities mixed into the sample. In all samples from 0 and 24 h, a significant compound (peak 1, Supplementary Figure S5) was detected at 17.668 min and displayed a characteristic mass fragment [M+] at *m*/*z* = 99, with major fragment ions at *m*/*z* = 43 and 152 (Figure 4A). Its molecular formula (C_13_H_24_O_2_) and molecular weight (213) were similar to those of DSF. The amount of DSF decreased with time, and a new compound (peak 2, Supplementary Figure S5) was detected at a retention time (RT) of 21.162 min. The new compound was similar to *trans*-2-dodecenoic acid, with *m*/*z* = 43 as the base peak (Figure 4B). Interestingly, this new compound disappeared with the complete degradation of DSF.

**FIGURE 5:**
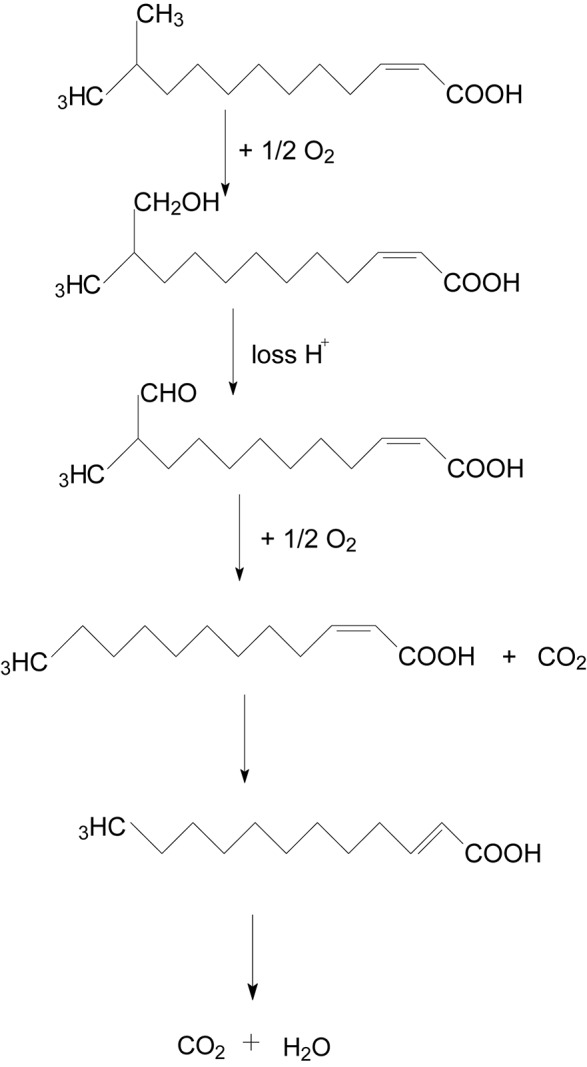
Proposed degradation pathway of DSF in *Acinetobacter lactucae* strain QL-1.

The degradation pathway of DSF in strain QL-1 is proposed based on the chemical structures of DSF (*cis*-11-methyl-2-dodecenoic acid) and the intermediate product (*trans*-2-dodecenoic acid) (Figure 5). Similar to oxidation of fatty acid, DSF degradation initiates as the enzyme catalyzes hydrogen oxidation on branched carbon atoms to hydroxyl, producing α-hydroxyl fatty acid. Hydroxyl fatty acid oxidizes and removes carboxyl groups to form fatty acids with one less carbon atom. The shortened carbon chain continues to undergo β-oxidation, and unsaturated fatty acids containing one double bond convert *cis* double bonds into *trans* double bonds by beta-oxidation. Eventually, DSF is degraded to carbon dioxide and water.

### Assessment of QL-1 for Biological Control of Disease Caused by *Xcc*

**FIGURE 6:**
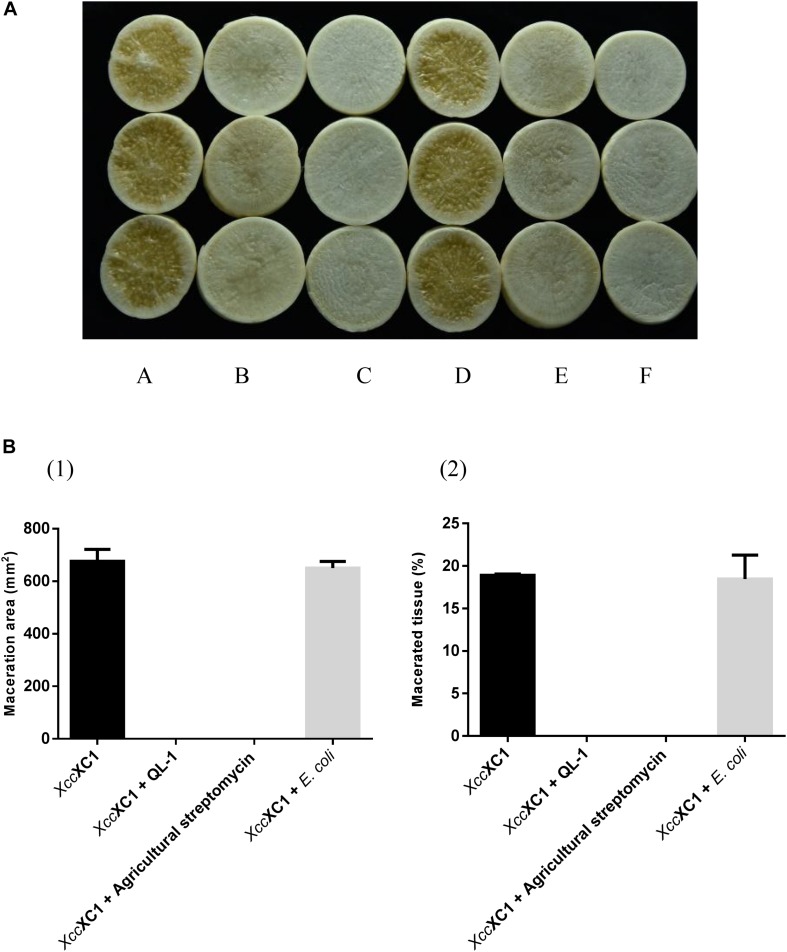
Biocontrol test of strain QL-1 against black rot disease on radish slices under laboratory conditions. **(A)** Panel a, XC1 alone on radish slices; panel b, XC1 + QL-1; panel c, XC1 + agricultural streptomycin; panel d, XC1 + *E. coli* DH5α; panel e, QL-1 alone. Panel f consists of untreated radish slices as a control. **(B)** Maceration area (1) and macerated tissue rate (2) in each treatment.

**FIGURE 7:**
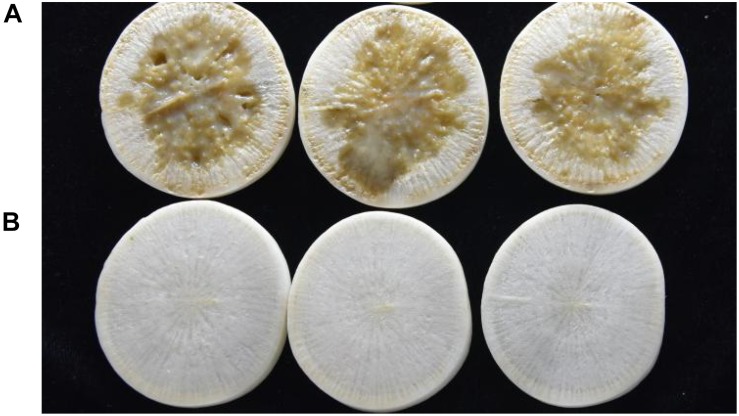
Preventive control test of strain QL-1 against black rot disease on radish slices under laboratory conditions. Panel **A** was only inoculated with XC1. Panel **B** was inoculated with QL-1 6 h before XC1 was added.

Strain QL-1 exhibited 100% biocontrol efficiency against *Xcc* on radish slices at a concentration of 1 × 10^9^ CFU mL^–1^ (Figure 6). Individual treatment of strain QL-1 substantially reduced disease severity caused by *Xcc*. Maceration was not observed in plant slices treated with a mixture of QL-1 and XC1 (Figure 6, panel b), similar to the treatment of agricultural STR and XC1 mixture (Figure 6, panel c). Plant slices solely treated with XC1 or the *E. coli* DH5α and XC1 mixture resulted in severe disease incidence (Figure 6, panels a,d; Figure 7A). Application of strain QL-1 on radish was safe (Figure 6, panel e). Disease symptoms were also not observed in preventive biocontrol tests (Figure 7B). These results indicated that strain QL-1 is a potent biological control agent against *Xcc* and can be used as a pre-infection preventive measure for plant protection.

### Biocontrol Efficiency of QL-1 Crude Enzymes Against *Xcc*

**FIGURE 8:**
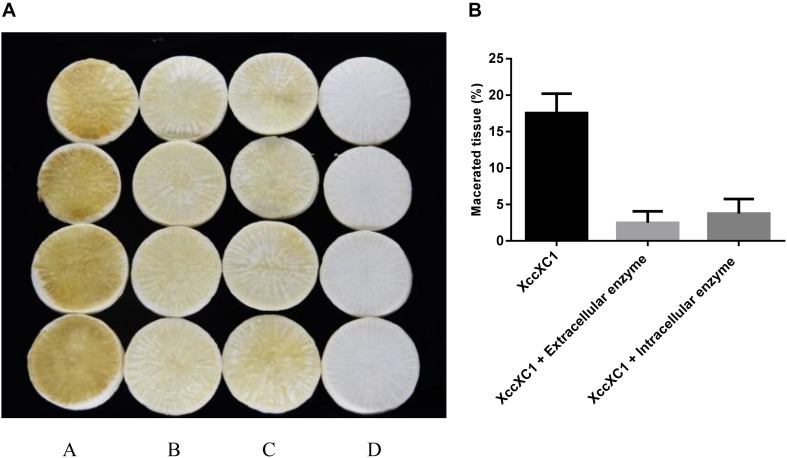
Preliminary biocontrol test of crude enzymes of QL-1 against black rot disease on radish slices under laboratory conditions. **(A)** Panel a, XC1 alone; panel b, XC1 + extracellular enzymes of QL-1; panel c, XC1 + intracellular enzymes of QL-1; panel d, untreated radish slices as a control. **(B)** Percentage of macerated tissue in each treatment.

Crude cell enzymes exhibited promising biocontrol efficiency against *Xcc*. Slight decay was observed in radish slices after treatment with extracellular and intracellular enzymes of isolate QL-1 (Figure 8A, panels b,c). However, the areas of macerated tissue (Figure 8B) were significantly reduced (*P* < 0.05) from 18.3 to 3.7%, and 18.3 to 4.3%, respectively, compared with individual XC1 treatment. The results indicated that crude enzymes of strain QL-1 significantly reduced the incidence of black rot, highlighting its potential against *Xcc* pathogenicity.

### Antimicrobial Susceptibility Test

Supplementary Figure S6 shows the antibiotic sensitivity of *A. lactucae* strain QL-1. Resistance of QL-1 reached 200 mg mL^–1^ against CM, 50 mg mL^–1^ against AMP, and 20 mg mL^–1^ against RIF and carboxybenzylpenicillin (CARB). Resistance to STR, KAN, GEN, and TET was noted to be <10 mg mL^–1^.

## Discussion

Plant diseases are the outcome of the synchronized activity of regulation networks, virulence factors, and infection processes of bacterial pathogens (Bzdrenga et al., 2017). Along with sensing plant signals and nutrient availability, QS also plays a vital role in the pathogenic cycle. QQ disrupts QS by degrading and interfering with signal generation or perception. This technique has evolved as a promising novel strategy for the prevention and control of QS-mediated bacterial infections (Mole et al., 2007). The plant pathogenic bacteria *Pseudomonas* spp., *Burkholderia* spp., *Dickeya* spp., *Pectobacterium* spp., *Erwinia* spp., and *Pantoea* spp. produce AHLs; *Pantoea* spp., *Erwinia* spp., and *Pectobacterium* spp. produce autoinducer-2 (AI-2), a furanosyl-borate diester; *Legionella* spp. and *Vibrio* spp. produce alpha-hydroxy-ketones; and *Xylella fastidiosa* and *Xanthomonas* spp. produce a wide variety of diffusible signal factors (DSFs) (Flavier et al., 1997; Chen et al., 2002; Kendall and Sperandio, 2007; Zhang et al., 2007; Mei et al., 2010; Churchill and Chen, 2011; Winans, 2011; Lee and Zhang, 2015; Ryan et al., 2015; Liu et al., 2017; Zhan et al., 2018). DSF is widely conserved in gram-negative bacterial pathogens (Deng et al., 2011; Zhou et al., 2017).

Interestingly, QQ enzymes can degrade most of these signaling molecules (Dong et al., 2000, 2004; Zhang et al., 2002; Carlier et al., 2003; Uroz et al., 2008; Krysciak et al., 2011). To date, only a few studies have reported the application of DSF-degrading bacteria against *Xcc*, but the production of DSF-degrading enzyme CarAB (a carbamoyl phosphate synthetase) has been reported in several *Pseudomonas* spp. (Shinohara et al., 2007; Newman et al., 2008). These reports verified QQ as an efficient disease prevention method in plants under laboratory conditions.

During this study, a DSF-degrading bacterium, QL-1, was isolated from soil, which completely degraded (100%) DSF and produced a major intermediate product (*trans*-2-dodecenoic acid). Alpha-oxidation is important in the catabolism of branched-chain fatty acids (Casteels et al., 2010) for the synthesis of hydroxy fatty acids. Alpha-hydroxy fatty acid can be further oxidized and decarboxylated to a fatty acid one carbon shorter than the original. Therefore, if an odd-chain-length compound is initially used, an even-chain-length acid is produced that can be further oxidized by β-oxidation (Quant and Eaton, 1999). Similarly, we speculated that hydrogen of the branched carbon atom in DSF was oxidized to hydroxyl fatty acid. Hydroxyl fatty acid was further oxidized to remove the carboxyl group, and fatty acids with one less carbon atom were formed. To facilitate beta-oxidation, the shortened carbon chain converted *cis* double bonds into *trans* double bonds, and, eventually, DSF was degraded to carbon dioxide and water without any persistent accumulative product (Figure 5). Therefore, we deduced that QL-1 may possess a complete metabolic pathway for DSF degradation and metabolism.

Morphological features, 16S rRNA gene analysis, and Biolog tests revealed the identity of the QL-1 strain to be *A. lactucae*, which is a novel species recently reported by Rooney et al. (2016). No literature is available about the potential biocontrol activity of *A. lactucae* against *Xcc*.

In addition to its DSF-degrading ability, this study also investigated the biocontrol potential of strain QL-1. Garge and Nerurkar (2017) reported an AHL-degrading isolate, Pls17, that caused tissue maceration in potato and therefore was not considered as a biocontrol agent. Not all bacterial species are capable of degrading signaling molecules, and a successful biocontrol agent should not produce detrimental effects on the host. During the current study, strain QL-1 had 100% biocontrol efficiency against *Xcc* without any side-effects. Similar to with a mixture of STR and XC1, tissue maceration was not observed after treatment with a mixture of QL-1 and XC1. The results indicated a significant reduction in black rot disease indices after treatment with strain QL-1, and preventive treatment completely inhibited disease symptoms. A slight decay on radish slices was observed after treatment with extracellular and intracellular enzymes of strain QL-1 and XC1. The results of the present study suggest that strain QL-1 and its crude enzymes are potent biological control agents. They can also be applied as a pre-infection preventive measure to control/attenuate black rot and other infectious diseases caused by DSF-dependent bacterial pathogens.

## Conclusion

In this study, a simple and efficient method was developed for the screening of highly active DSF-degrading microorganisms. A novel bacterial isolate, *A. lactucae* QL-1, possessing excellent DSF-degrading activity, was identified. Strain QL-1 utilized DSF as the sole carbon source for its growth and rapidly degraded DSF. Experiments confirmed that DSF-degrading bacterium QL-1 and its crude enzymes substantially reduced the disease severity of *Xcc* and hence could also be used in the pre-infection period to control and prevent DSF-mediated bacterial infections. These findings establish strain QL-1 and its crude enzymes as potential biocontrol agents against infectious diseases caused by DSF-dependent bacterial pathogens. The application of efficient biocontrol agents will minimize the use of chemical pesticides and reduce their harmful effects. An in-depth study of DSF degradation-related genes and enzymes will provide a clear understanding of the pathway of DSF molecules in the natural microenvironment. Recently developed omics tools can facilitate the complete analysis of the mechanisms of QQ strains, which will help in the reduction of economic losses due to crop diseases.

## Data Availability Statement

The datasets generated for this study are available on request to the corresponding author.

## Author Contributions

SC and LZ designed the experiment. TY and TZ performed the experiment. TY, TZ, and XF analyzed the data. TY, TZ, XF, PB, and SC wrote the manuscript.

## Conflict of Interest

The authors declare that the research was conducted in the absence of any commercial or financial relationships that could be construed as a potential conflict of interest.
