# Supplementary material for: Acinetobacter lactucae Strain QL-1, a Novel Quorum Quenching Candidate Against Bacterial Pathogen Xanthomonas campestris pv. campestris
Source: Front Microbiol. 2019 Dec 17;10:2867. doi: 10.3389/fmicb.2019.02867 (PMC6929412; doi:10.3389/fmicb.2019.02867)
Supplement: Supplementary file 1 [file Data_Sheet_1.pdf]

Supplemental files

Acinetobacter lactucae strain QL-1, a novel quorum quenching candidate against bacterial pathogen *Xanthomonas campestris* pv. *campestris*

Tian Ye[1,2#], Tian Zhou[1,2#], Xinghui Fan[1,2], Pankaj Bhatt[1,2], Lianhui Zhang[1,2], Shaohua Chen[1,2*]

[1]State Key Laboratory for Conservation and Utilization of Subtropical Agro-bioresources, Guangdong Province Key Laboratory of Microbial Signals and Disease Control, Integrative Microbiology Research Centre, South China Agricultural University, Guangzhou 510642, P.R. China;

[2]Guangdong Provincial Laboratory of Lingnan Modern Agricultural Science and Tech nology, South China Agricultural University, Guangzhou 510642, P.R. China

Running title: Efficacy of *A. lactucae* QL-1 in DSF degradation

[#] Both authors contributed equally to this work.

[*]**Correspondence:**

Shaohua Chen

shchen@scau.edu.cn

Table S1 Biochemical characteristics of strain QL-1

Substrate	Result
D – glucose	+
D – glucose	+
D – fucose	+
D – galactose	−
D – fructose	−
Sucrose	−
Glycerin	−
D – mannitol	−
L – alanine	+
L – arginine	+
Amino acetyl-L-proline	−
L – lactic acid	+
Propionic acid	+

Note: +, positive; –, negative.

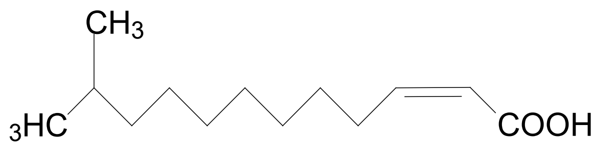

Fig. S1. Chemical structure of DSF

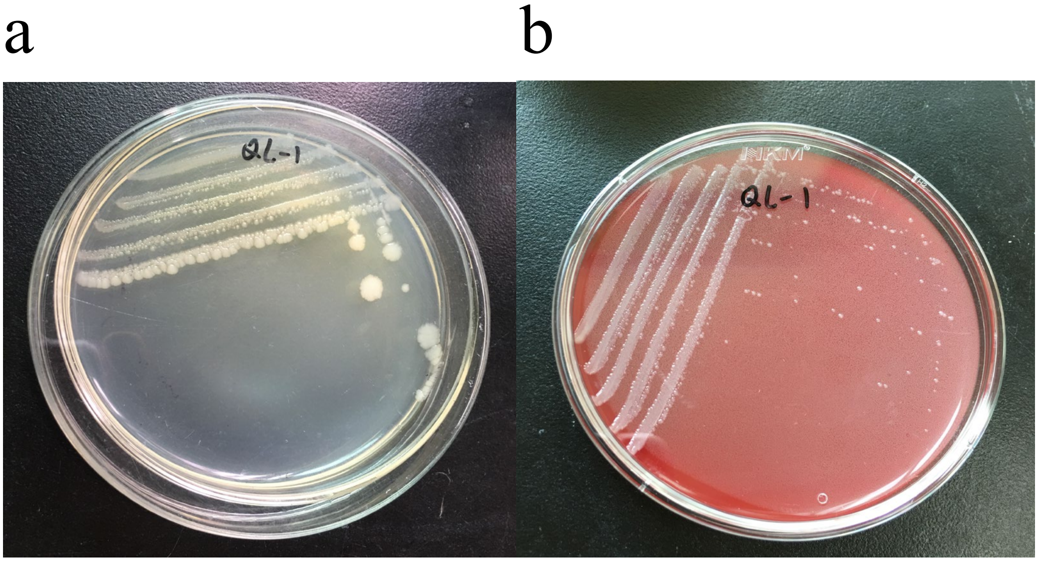

Fig. S2. Colony characteristics of strain QL-1 on LB (a) and blood (b) plates

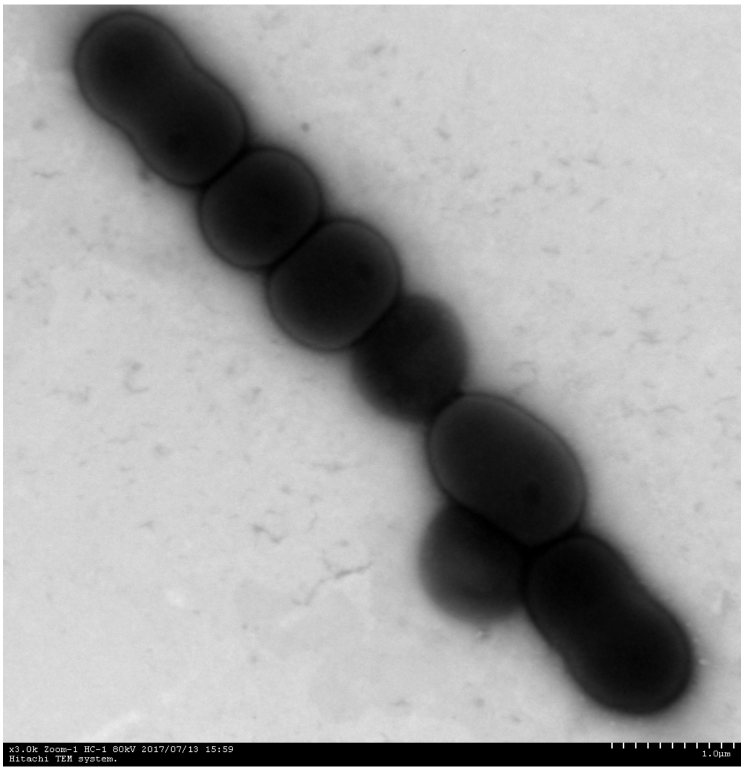

Fig. S3. Morphological characteristics of strain QL-1 imaged with Hitachi TEM System (3000×)

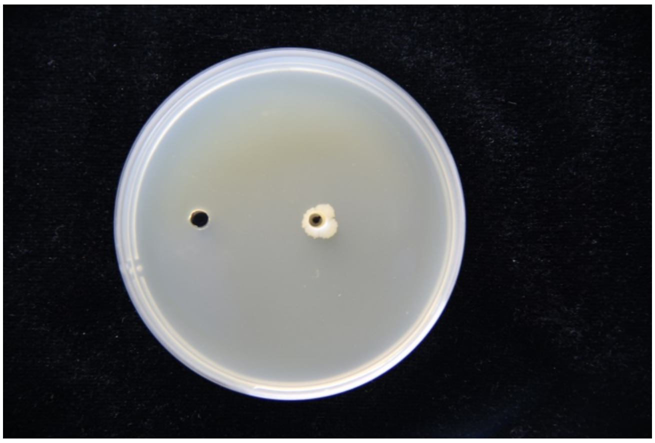

Fig. S4. Strain QL-1 showed no antagonistic activity against *Xcc*. Holes punched in LB plate containing culture suspension of *Xcc*, the left was un-inoculated LB and right was culture suspension of strain QL-1.

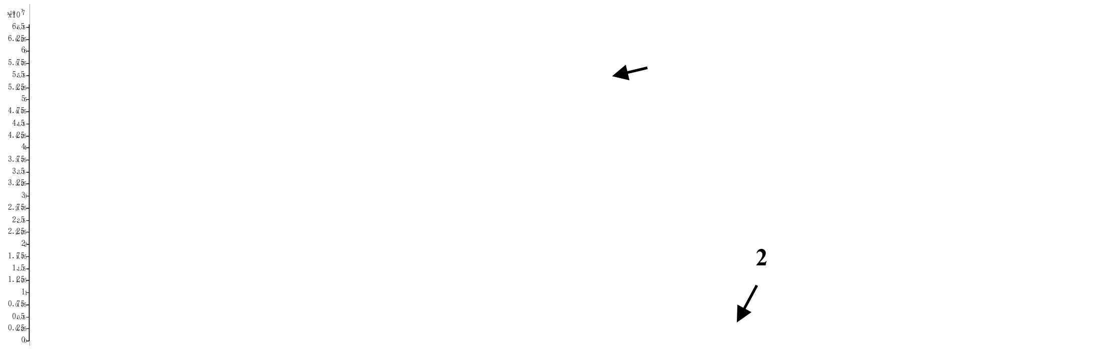

Fig. S5. Elution behavior of 5 mmol·L^{-1} DSF and its degradation products on GC column.

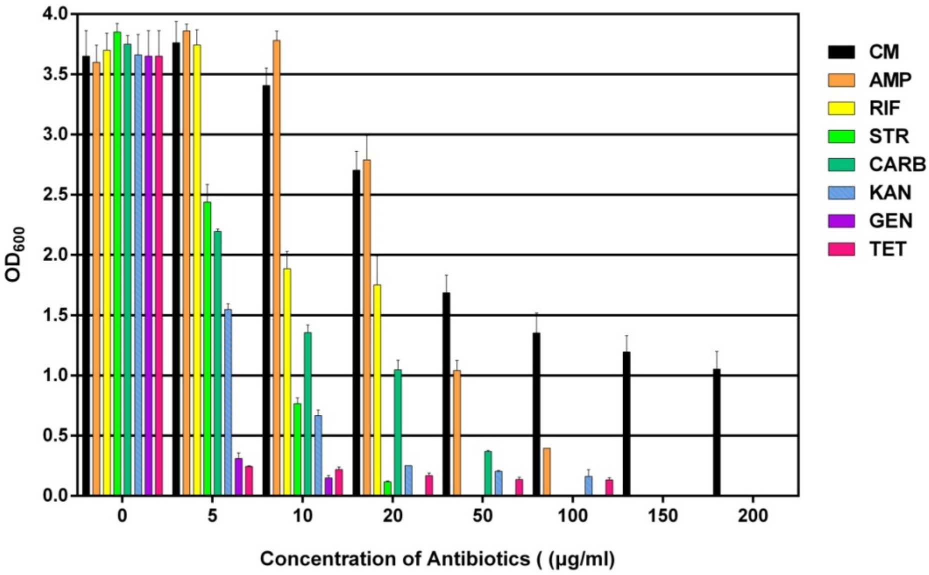

Fig. S6. Antibiotic sensitivity of Acinetobacter lactucae strain QL-1. Resistance of the strain QL-1 to chloramphenicol (CM) reached 200 mg·mL^{-1}, resistance to ampicillin (AMP) reached 50 mg·mL^{-1}, resistance to rifampicin (RIF) and carboxybenzylpenicillin (CARB) reached 20 mg·mL^{-1}, and resistance to streptomycin (STR), kanamycin (KAN), gentamicin (GEN) and tetracycline (TET) was less than 10 mg·mL^{-1}.
